# Dissociative Electron Attachment to 5-Iodo-4-thio-2′-deoxyuridine: A Potential Radiosensitizer of Hypoxic Cells

**DOI:** 10.1021/acs.jpclett.3c02219

**Published:** 2023-09-28

**Authors:** Muhammad Saqib, Eugene Arthur-Baidoo, Farhad Izadi, Adrian Szczyrba, Magdalena Datta, Sebastian Demkowicz, Janusz Rak, Stephan Denifl

**Affiliations:** † Institut für Ionenphysik und Angewandte Physik, Universität Innsbruck, Technikerstraße 25, A-6020 Innsbruck, Austria; ‡ Center for Molecular Biosciences Innsbruck, Universität Innsbruck, Technikerstraße 25, A-6020 Innsbruck, Austria; § Laboratory of Biological Sensitizers, Department of Physical Chemistry, Faculty of Chemistry, University of Gdańsk, Wita Stwosza 63, 80-308 Gdańsk, Poland; ∥ Department of Organic Chemistry, Faculty of Chemistry, Gdańsk University of Technology, Narutowicza 11/12, 80-233 Gdańsk, Poland

## Abstract

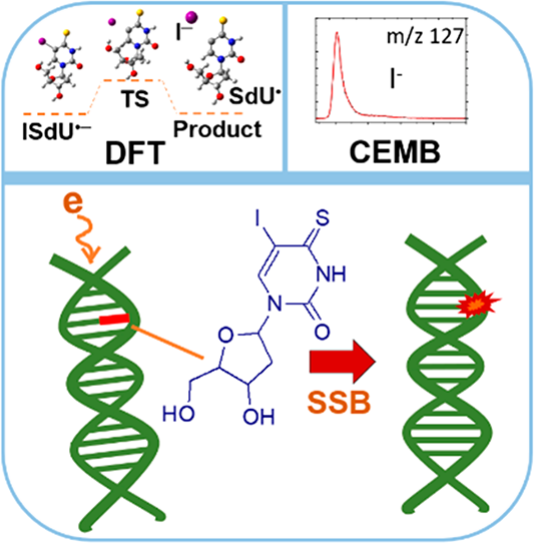

In the search for effective radiosensitizers for tumor cells, halogenated uracils have attracted more attention due to their large cross section for dissociation upon the attachment of low-energy electrons. In this study, we investigated dissociative electron attachment (DEA) to 5-iodo-4-thio-2′-deoxyuridine, a potential radiosensitizer using a crossed electron-molecule beam experiment coupled with quadrupole mass spectrometry. The experimental results were supported by calculations on the threshold energies of formed anions and transition state calculations. We show that low-energy electrons with kinetic energies near 0 eV may effectively decompose the molecule upon DEA. The by far most abundant anion observed corresponds to the iodine anion (I^–^). Due to the associated bond cleavage, a radical site is formed at the C5 position, which may initiate strand break formation if the molecule is incorporated into a DNA strand. Our results reflect the conclusion from previous radiolysis studies with the title compound, suggesting its potential as a radiosensitizer.

It is known that low-energy electrons are released as secondary particles from the passage of primary high-energy radiation through biological matter like cells.^1,2^ While being ballistic particles, those low-energy electrons may induce DNA damage via bond rupturing and the creation of neutral or ionic radicals that can induce further damage over time.^3,4^ At electron energies below ∼15 eV, dissociative electron attachment (DEA) is an elemental mechanism for this damage. Upon electron attachment, initial temporary negative ions (TNIs) are formed. If the TNI state is dissociative in the Franck–Condon region and its lifetime is sufficiently long with respect to autoionization, the decay of the TNI can lead to fragment anion and neutral(s) formation. It was shown that low-energy electrons may induce strand breaks and/or other damage in the biomolecular films of DNA^5^ and DNA origami triangles.^6^ Solution phase experiments also demonstrated that electrons, while still being quasi-free, may induce bond cleavage in DNA constituents.^7,8^ In contrast, electrons entering the prehydrated or hydrated stage do not seem to be effective in DEA to DNA nucleobases in solution.^9^

In recent years, investigations with isolated or microhydrated DNA constituents in the gas phase substantially contributed to the understanding of the dynamics of electron attachment to biomolecular systems.^10−12^ This knowledge is also essential in the search for new molecules, which should enhance the effects of ionizing radiation in tumor cells.^13−15^ Such so-called radiosensitizers may be designed so that they are particularly prone to low-energy electron attachment.^16,17^ DEA could be then a mechanism that is exploited for the generation of species (like free radicals) damaging the DNA in tumor cells.^18−21^ To study the basic electron attachment properties of potential radiosensitizers, crossed electron-molecule beam (CEMB) experiments were carried out.^22−25^ There was early interest in DEA to halogenated uracils.^26^ The incorporation of these modified uracils into native DNA should enhance radiation-induced cell killing due to their strong electrophilic properties and low cytotoxicity toward cancer and normal cells.^27^

Being susceptible to electron-induced decompositions upon electron attachment, the relevant DEA reaction corresponds to the cleavage of the C5 bond, forming a halogen anion and leaving behind the neutral uracil-yl radical. Indeed, the DEA study by Abdoul-Carime et al.^26^ demonstrated the desired outcome of the DEA reactions in halouracils because the corresponding halogen anions were observed as abundant reaction products. Among the studied halouracils, 5-iodouracil (IU) showed favorable DEA properties.^26^ Its nucleoside derivative, 5-iodo-2′-deoxyuridine (IdU), was first synthesized in the late 1950s to serve as an antitumor drug but was then more often used as an antiviral drug in the treatment of herpetic keratitis.^28^ Though IdU was tested as a radiosensitizer in the treatment of high-grade gliomas, it found no standard practical applications in radiation therapy.^29^ A more recent phase 0 trial study demonstrated the potential of oral 5-iodo-2-pyrimidinone-2′-deoxyribose (IPdR), a prodrug of IdUrd, for the radiation treatment of advanced malignancies.^30^

To have alternative potential radiosensitizers operating on the DEA mechanism, Rak and co-workers proposed and synthesized several other C5-substituted uracil derivatives^27^ that were studied both in the gas phase with respect to DEA, supported by computational supporting tools, and in the solution phase.^25,31^ Recently, the radiosensitizing properties of 5-iodo-4-thio-2′-deoxyuridine (ISdU) and 5-bromo-4-thio-2′-deoxyuridine (BrSdU) in the solution phase were investigated.^32,33^ ISdU and BrSdU were previously proposed as potential photosensitizers.^34,35^ Due to the presence of a sulfur atom in the molecules, they absorb in the UVA region (∼350 nm) far behind the maximum of DNA absorption (∼260 nm), and irradiation of DNA labeled with these nucleoside modifications leads to interstrand cross-links and DNA strand breaks.^34^ ISdU and BrSdU are 2′-deoxyuridine derivatives with the oxygen at C4 and the hydrogen at C5 positions substituted by the sulfur atom and the corresponding halogen atoms, respectively. The potential of ISdU as an effective radiosensitizer was recently demonstrated via studies involving clonogenic assays and steady state radiolysis with the OH^•^ radical scavenger.^32,33^ In contrast, BrSdU did not show promising radiosensitizing properties.^32^ This different outcome was explained by the longer lifetime of the BrSdU radical anion, which allows the efficient protonation and quenching of DEA.^32^

In this paper, we report our findings on the electron-induced dissociation within the ISdU molecule (C_9_H_11_N_2_O_4_SI) upon electron attachment. Experimentally, we find that the formation of halogen anion I^–^ corresponds to the predominant process. We computationally describe the reaction pathways of all four fragment anions found within the detection limit of the apparatus we used. These results for ISdU in the gas phase turn out to be in line with the properties of ISdU found in the previous radiolysis studies with this compound, confirming the strong radiosensitizing potential of the studied system. However, a huge amount of work is still needed to introduce ISdU into clinical practice. In particular, positive animal tests are required to initiate clinical trials. The sooner these in vivo studies are carried out, the better the chance of introducing ISdU into clinics. Therefore, rapid dissemination of our results is well justified.

In our experiment, a molecular beam of ISdU was crossed with a well-defined electron beam to study the fragmentation yield versus the incident electron energy in the energy range from ∼0 to 10 eV (for details of the experiment see section S.1 of the Supporting Information)

We observed four fragment anions at *m*/*z* 253, 127, 126, and 33 within the detection limit of the experiment. The anion efficiency curves for the observed fragment anions upon DEA to ISdU are shown in Figure 1a–d. The respective plots show the region of interest in which resonance ion yield was found. Figure 1 also indicates the corresponding cumulative fit of the observed peaks. The derived peak maxima are summarized in Table 1.

**Figure 1:**
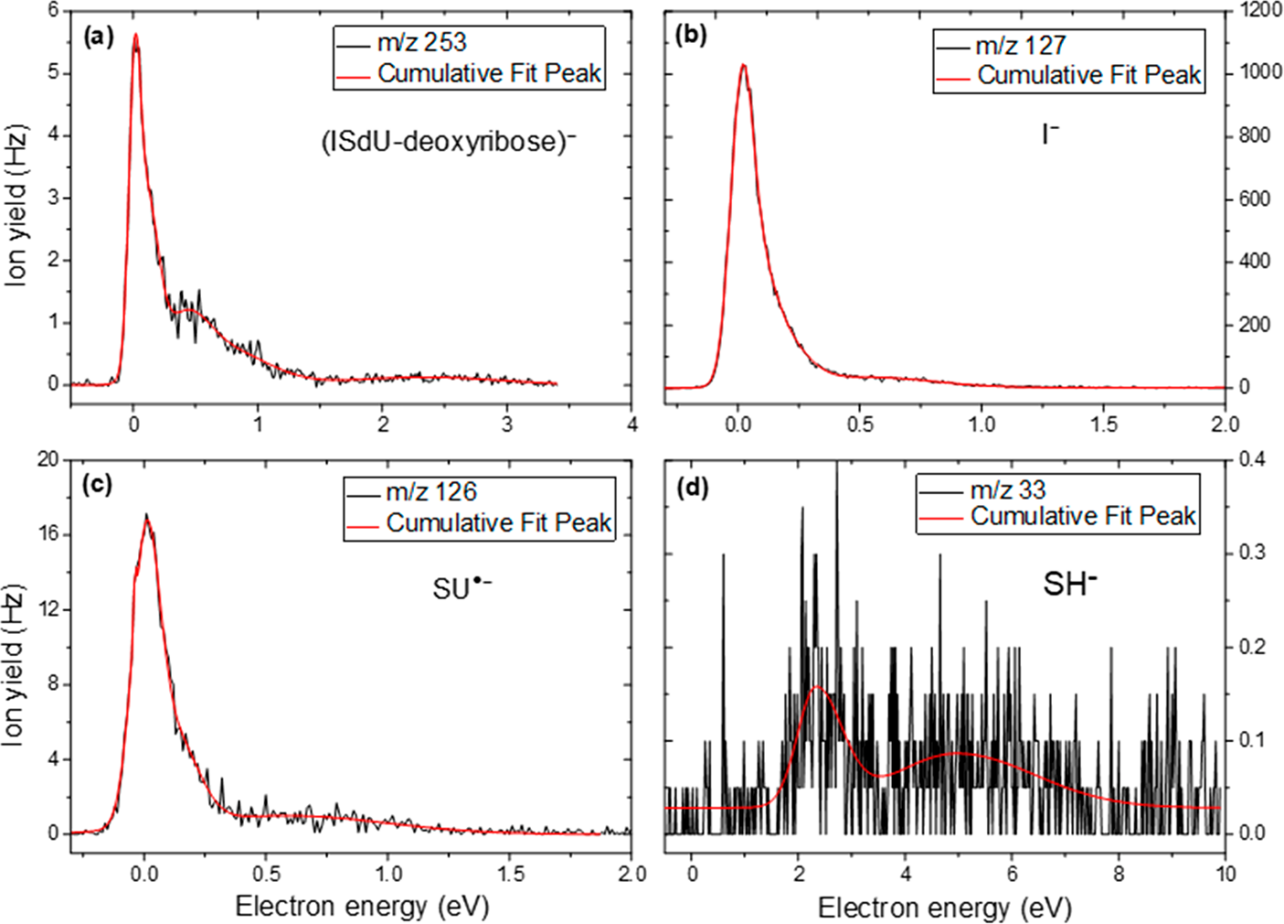
Anion efficiency curves of the fragment anions formed upon electron attachment to ISdU: (a) (ISdU-deoxyribose)^−^, (b) I^–^, (c) SU^•–^, and (d) SH^–^. The red line corresponds to the cumulative fit of the measured ion yield.

**Table 1:** Summary of the Observed Fragment Anions in Terms of Masses, Structural Assignments, and Their Corresponding Maxima on the Anion Efficiency Curves, as Well as the Experimental and Calculated Thresholds (Δ*E*_0_)

		maxima of peak positions (eV)				threshold (eV)	
mass (units)	anion	1	2	3	4	expt (383 K)	calcd (Δ*E*_0_)
253	(ISdU-deoxyribose)^−^	∼0	0.4	0.8	2.3	∼0	–0.35
127	I^–^	∼0	0.5	–	–	∼0	–0.38
126	SU^•–^	∼0	0.1	0.4		∼0	0.00^a^
							0.49^b^
33	SH^–^	2.2	5.5	–	–	1.6	1.70

a Reaction 3a.

b Reaction 3b (see Figure 3).

The experimentally found threshold for each fragment anion (derived by a simple method introduced in ref (23)) is also listed in Table 1 and compared with the computationally obtained thresholds.

Experimentally, we did not find any signal of the intact parent anion (mass of 370 units) within the detection limit of the apparatus. Although exceptions exist,^36^ parent anions are usually detected in mass spectrometric experiments most abundantly at the electron energy near 0 eV.^37^ A positive electron affinity (corresponding to the situation in which the ground state of the anion is energetically below that of the neutral molecule) of sufficiently high value is required for a lifetime on the order of at least microseconds, allowing detection by mass spectrometry. The calculated adiabatic electron affinity of ISdU at the M06-2X/DGDZVP++ level is ∼1.43 eV (see Figure 2), which is close to that very recently reported for BrSdU (for details of the computational methods see section S.2).^38^

**Figure 2:**
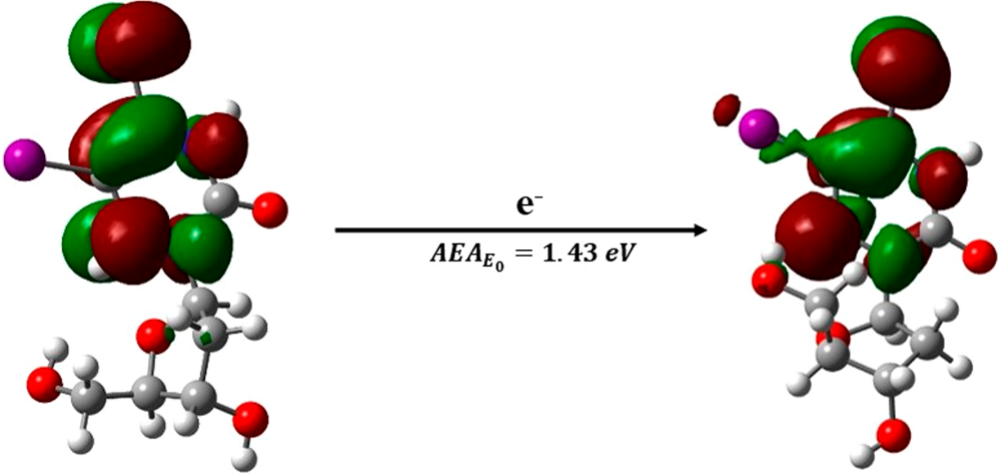
Adiabatic electron affinity (AEA) in terms of zero-point energy-corrected total energy calculated at the M06-2X/DGDZVP++ level. The following color codes were used to indicate particular atoms: white for H, gray for C, blue for N, red for O, yellow for S, and violet for I. LUMO or SOMO orbitals were superimposed on the structures of the ISdU neutral form (left) and anion radical (right).

Thus, the parent anion of ISdU decays by either fast spontaneous emission of the excess electron or, more likely, molecular bond cleavages (DEA) (see the discussion below). To the best of our knowledge, no previous work on electron attachment to the IdU in the gas phase has been published. In electron attachment to bromouridine (BrdU) in the gas phase, a parent anion could be obtained with our crossed beam setup.^39^ Abdoul-Carime et al. reported the formation of a stable parent anion upon electron attachment to IU, as well.^26^ Interestingly, the 0 eV peak of their parent anion yield was just a minor feature next to a sharp peak at 0.5 eV and a rather broad feature at 1.3 eV. Because dissociation channels were also open at these electron energies, they pointed out that different electronic states may be involved for the dissociative channels and the stabilized anion. A purely dipole-bound anion was considered, but the authors raised some doubts about its lifetime toward autodetachment.^26^ More recently, it was also shown that dipole-bound anions are effective doorway states for DEA.^40,41^ A similar situation of a detectable parent anion with strong competition by dissociation channels near 0 eV was also found for other halouracils,^26^ except fluorouracil.^42^

Instead of the formation of a long-lived parent anion, the formation of DEA to ISdU results in the formation of four fragment anions, which are due to the cleavage of bonds in the uracil moiety as well as the C–N glycosidic bond. Figure 3 shows the different pathways leading to the anions observed for DEA to ISdU.

**Figure 3:**
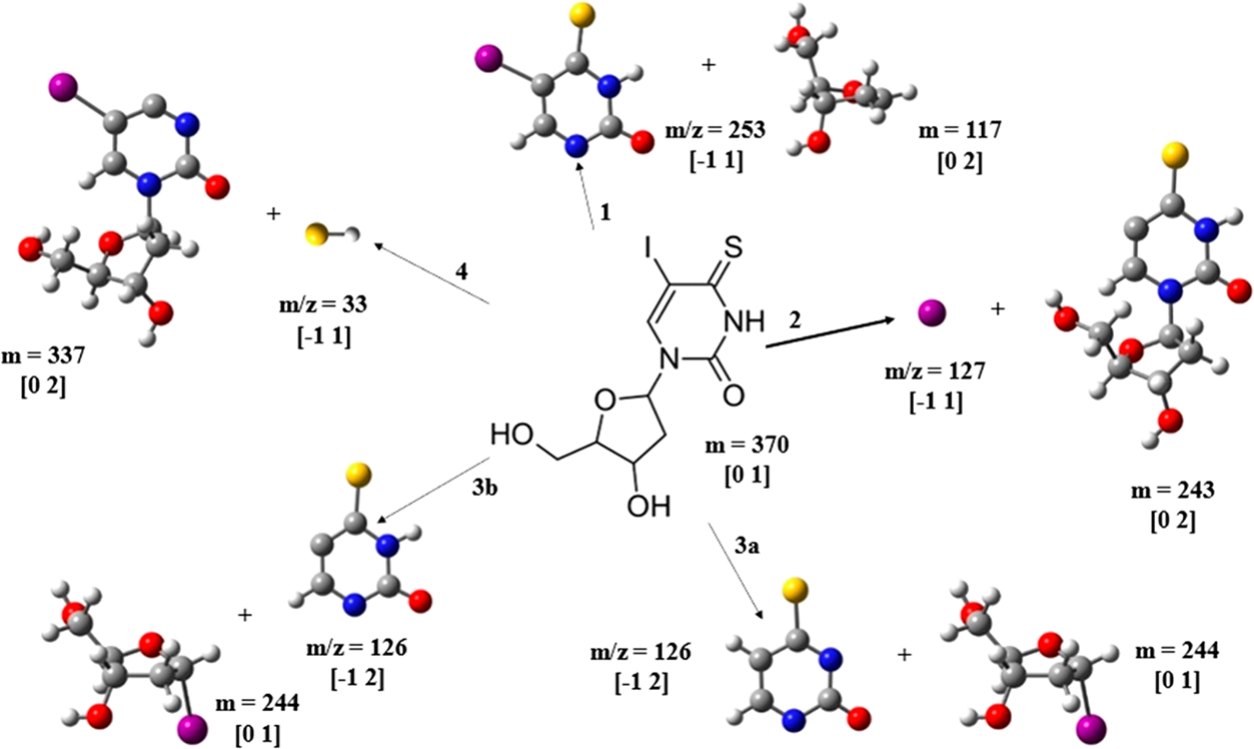
Dissociation pathways in the ISdU molecule upon low-energy electron attachment. The experimentally detected fragments are denoted with masses of *m*/*z* 253 (1), 127 (2) 126 (3a and 3b), and 33 (4). The charge and multiplicity are shown in brackets for each structure. The following color codes were used to indicate particular atoms: white for H, gray for C, blue for N, red for O, yellow for S, and violet for I.

Figure 3 illustrates (the addition of the electron is not shown) the anions and their corresponding neutrals after the dissociation process. The heaviest fragment anion was obtained at *m*/*z* 253, which can be associated with a single bond cleavage. The anion is formed after the C–N glycosidic bond cleavage, which leaves the deoxyribose component as the neutral fragment, as depicted in reaction 1 of Figure 3. The DEA reaction equation can be written as
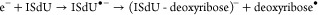
1

This computational result at the M06-2X/DGDZVP++ level of theory predicts a reaction energy of −0.35 eV for neutral ISdU.

The respective activation energy [ISdU^•–^ → (ISdU-deoxyribose)^−^ + deoxyribose^•^] for this reaction amounts to 1.07 eV (see the TS_253 structure in Figure 4), which is by 0.36 eV smaller than the exoergic effect related to electron attachment [1.43 eV (see the red broken line in Figure 4)]. Hence, this reaction should be triggered, as is actually observed (Table 1), by electrons with a kinetic energy of 0 eV. For (ISdU-deoxyribose)^−^, the anion efficiency curve exhibits a major resonance energy at ∼0 eV (see Figure 1a). Thus, the experimental ion threshold observed agrees well with the predicted one. Other less intense inherent peaks can be observed at 0.4 eV and in the extended tail near ∼0.8 eV. Furthermore, a peak is observed at 2.3 eV with a broad spread between 1.5 and 3.5 eV. It is worth mentioning that, even though the C–N bond cleavage is the channel with the heaviest mass fragment for the DEA reaction, the intensity of this anion is 2 orders of magnitude lower than that of the most intense fragment anion.

**Figure 4:**
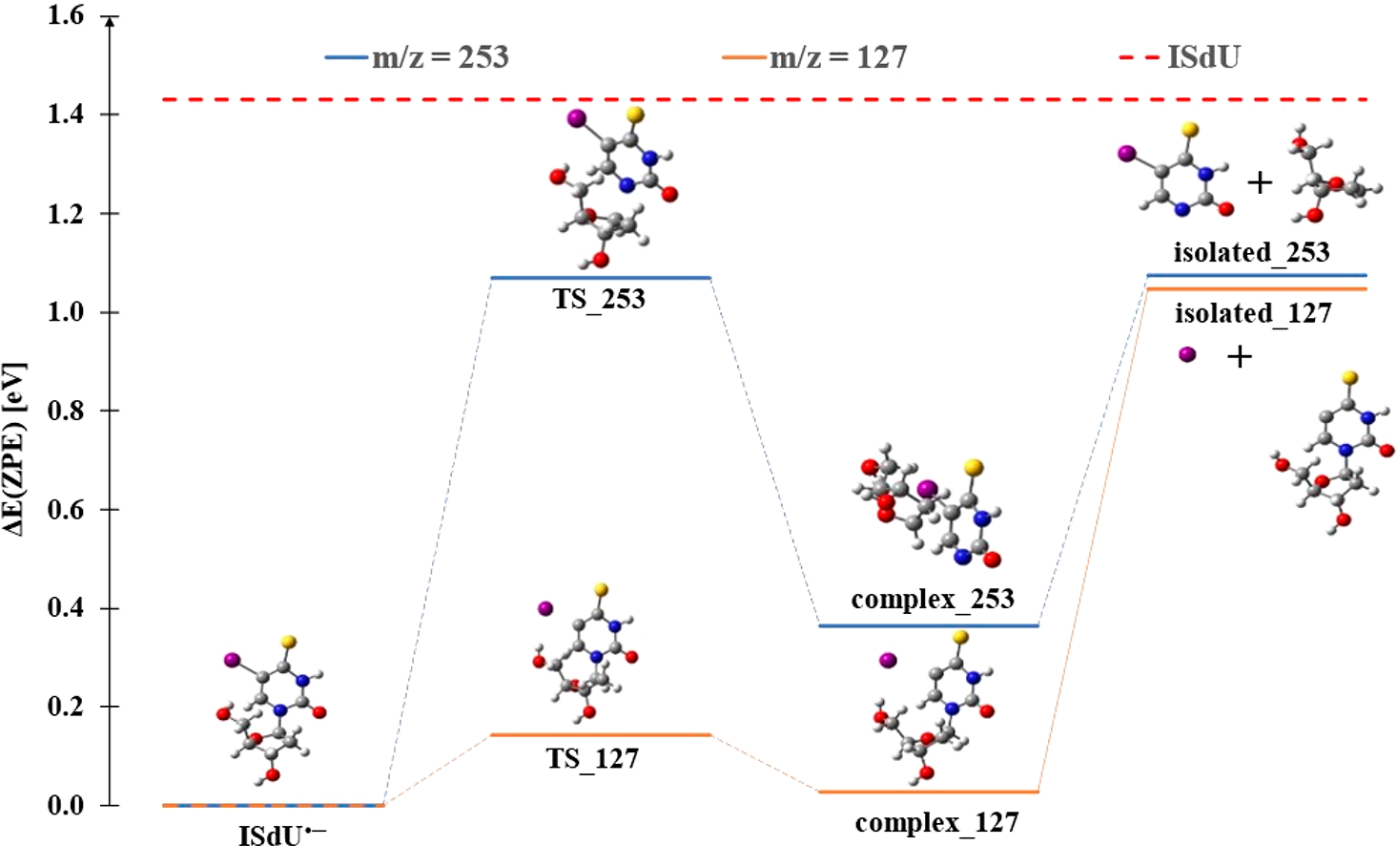
Single bond cleavage pathways, leading from the ISdU^•–^ anion radical to the anionic products at *m*/*z* 253 and 127. TS_*x*, complex_*x*, and isolated_*x*, where *x* refers to the value of *m*/*z*, stand for the transition state, product complex, and isolated monomers, respectively.

The second heaviest fragment anion formed upon DEA to ISdU was observed at *m*/*z* 127. This anion is by far the most abundant fragment upon electron attachment to ISdU. As suggested by reaction 2 of Figure 3, its formation involves single bond cleavage in the uracil moiety at the C5 position leading to the formation of the iodide anion
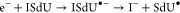
2

The formation of the halogen anion has been observed in DEA to all halogenated uracil derivatives studied.^26,42^ It has been shown that generally, the C–I bond is the weakest one between carbon and halogens^43^ and therefore can easily undergo bond homolysis. The current DEA results on ISdU in the gas phase are in line with the results obtained from the steady state radiolysis that reported the formation of the SdU^•^ as one of the main dissociative products following the loss of the iodide anion.^33^ In DEA studies with halouracils, which included 5-iodouracil (IU), the reaction pathway leading to the formation of I^–^ was reported as the most abundant one, see ref (26). Their result also showed that the intensity of its formation was notably ∼2–3 orders of magnitude larger than those of other formed anions such as the parent anion and the negatively charged dehalogenated derivative.^26^ A similar intensity difference can be observed in our experiment. Our result indicates, thus, the main radiosensitization mechanism of ISdU at the cellular level. Namely, after its enzymatic incorporation into DNA and the attachment of a solvated electron, the reactive thiouracil-5-yl radical is formed, which induces a DNA strand break. Hence, this finding suggests that ISdU should be administered well before actual irradiation during radiotherapy. A similar conclusion was drawn from radiotherapy studies on oxaliplatin in mice xenografts.^44^ Specifically, it was demonstrated that the strongest radiosensitizing effect occurs when the oxaliplatin concentration in DNA reaches the maximum, i.e., after administration of the drug for 48 h. Hence, the mechanistic information is crucial for further in vivo studies and justifies, similarly to the necessity of doing animal experiments, a rapid publication of the current paper. Finally, the description presented above clearly demonstrates that ISdU can radiosensitize cells only under hypoxia. Indeed, oxygen, at a relatively high concentration under normoxia (1.5 × 10^–3^ M),^45^ competes with ISdU for solvated electrons forming the O_2_^–^ radical, which is unreactive toward DNA. The anion efficiency shown in Figure 1b is characterized by a dominant (slightly asymmetric) peak at 0 eV. The predicted thermodynamic threshold for this channel is found to be −0.38 (Table 1). The activation energy for the cleavage of the C–I bond in ISdU^•–^ is small and amounts to 0.14 eV (see the TS_127 structure in Figure 4), showing that the excited ISdU anion formed after the electron attachment process possesses an excess energy of 1.29 eV above the level of the transition state. Hence, the activation barrier can be easily overcome, and isolated monomers, i.e., I^–^ + SdU^•^, form, which agrees pretty well with the experimental peak at ∼0 eV. As Figure 1b shows, another feature at 0.5 eV leads to the tail of the main peak. Though the yield of this feature at 0.5 eV is just ∼4% of the 0 eV peak, it is significant compared to the other fragment anions observed.

One can argue that because the findings presented above correspond to a situation of the isolated ISdU interacting with the excess electron, they may not hold for ISdU incorporated into DNA, as the double helix influences the formation of TNI. It is, however, worth noticing that our previous in vitro studies on ISdU confirm the radio sensitizing properties of the modified nucleoside against breast cancer cells.^33^ Thus, also being a part of DNA ISdU seems to be prone to DEA.

In addition to the fragmentation discussed so far, we observed a fragment anion at *m*/*z* 126, SU^•–^, which forms via only multiple bond cleavages and molecular rearrangement. One pathway includes an initial cleavage of the C–N glycosidic bond between the ISU and the deoxyribose moieties in the ISdU^•–^ anion (see the TS_253 structure in Figure 5) leading to complex_253 (see Figure 5) and is subsequently followed by the loss of the iodine atom from ISU^•–^ (see the TS1_126b structure in Figure 5). After the ISU anion is left, the neutral iodine atom is computationally predicted to reattach itself to the deoxyribose group. The overall reaction leading to the formation of the SU^•–^ anion may be shown by reactions 3a and 3b in Figure 3
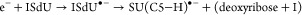
3a
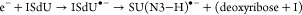
3b

**Figure 5:**
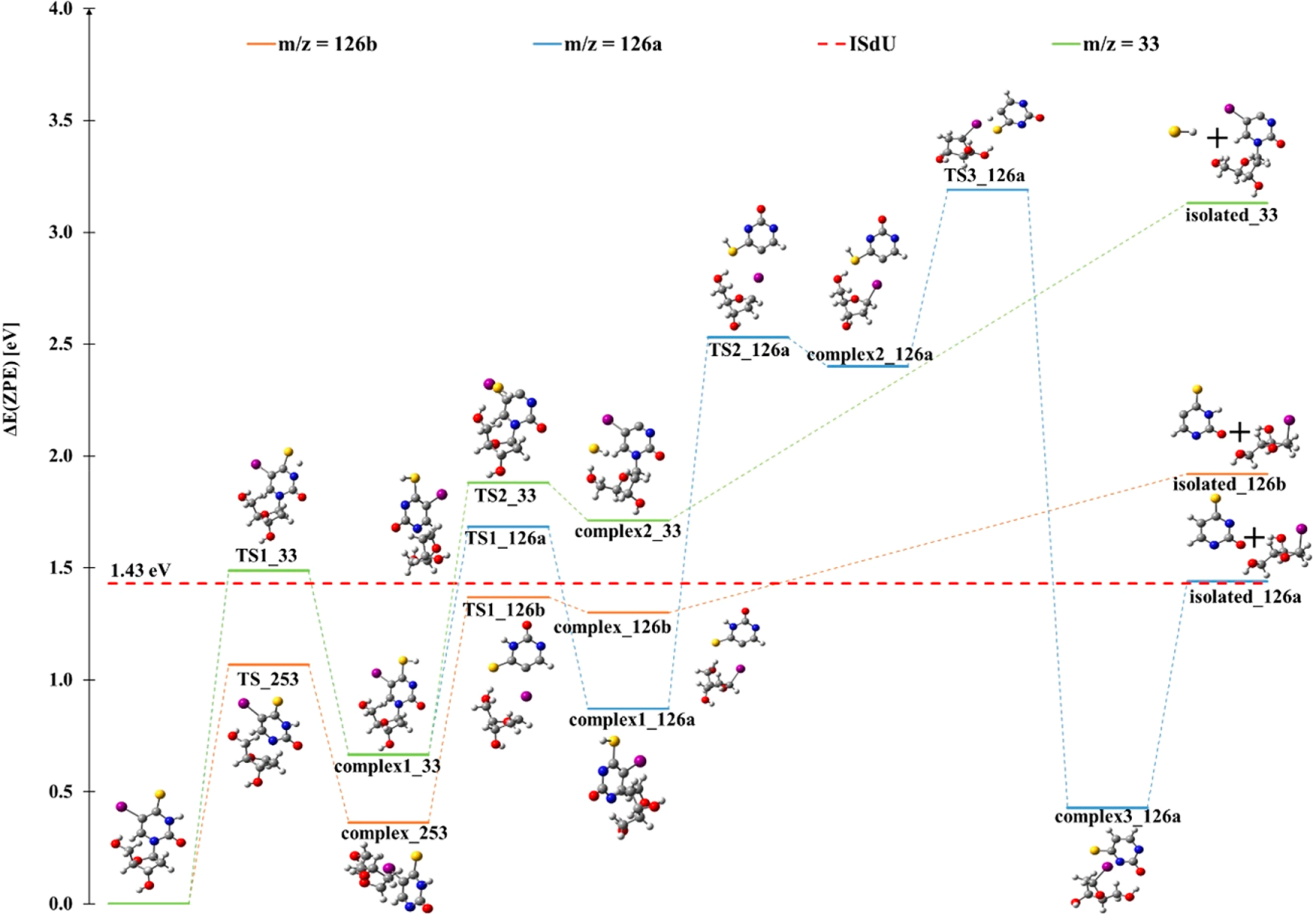
Multiple bond cleavage pathways, leading from the ISdU^•–^ anion radical to the anionic products at *m*/*z* 126 and 33. For the meaning of 126a and 126b, see Figure 1. TS*n*_*x*, complex*n*_*x*, and isolated*n*_*x*, where *n* is equal to 1 or 2 and *x* refers to the value of *m*/*z*, stand for the transition state, product complex, and isolated monomers, respectively.

In the latter process, a tautomer of SU^•–^ is formed in which the proton is bound to the N3 site, SU(N3–H)^•–^, while in the former reaction, the proton resides at the C5 position, SU(C5–H)^•–^. At first glance, reaction 3a may be suspected of being responsible for the formation of the SU^•–^ anion because its calculated threshold agrees with the experimental one (see the isolated_126a structure in Figure 5). However, as indicated by the structures of TS2_126a, complex2_126a, and TS3_126a (Figure 5), the calculated barriers significantly exceed the observed threshold. Thus, reaction 3b rather than reaction 3a leads to the experimentally observed SU^•–^. The calculated threshold for reaction 3b amounts to 0.49 eV, as indicated by Table 1 and Figure 5 (see the isolated_126b structure), but one should note that the respective product complex, complex2_126b, is 0.13 eV below the level of neutral ISdU (Figure 5), which means that it should be produced by 0 eV electrons. Assuming now that this weakly bound [0.62 eV (see Figure 5)] vdW complex is separated into components due to a hot band transition,^46^ the SU^•–^ anion is released. From the current experimental data shown in Figure 1c, the anion exhibits a main feature at ∼0 eV with a shoulder at ∼0.1 eV. The tail is further extended by a weakly abundant broad peak, with its maximum near 0.4 eV.

In this study, we also obtained very weak ion yields for the fragment anion at *m*/*z* 33, which may be assigned to SH^–^ formed in the reaction
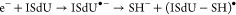
4

The formation of SH^–^ from ISdU is possible only if there is the attachment of the nearest hydrogen atom to the sulfur atom via proton transfer (structure TS1_33 in Figure 5) followed by the subsequent cleavage of the C–S bond as shown in reaction 4 of Figure 3 and transition state TS2_33 in Figure 5. At the M06-2X/DGDZVP++ level of theory, a reaction energy of 1.70 eV for the formation of SH^–^ was predicted (Table 1 and isolated_33 in Figure 5). Figure 1d shows the anion yield curve for SH, exhibiting two peaks at 2.2 and ∼5.5 eV. The experimental onset of 1.6 eV agrees with the theoretically determined threshold of 1.70 eV in view of the finite statistics of the experimental data.

From the results presented here, it is quite obvious that the deoxyribose moiety acts more as a spectator in the initial electron attachment, which is a behavior commonly suggested for more complex DNA networks.^10^ It should be noted that in their DEA study with IU, Abdoul-Carime et al. reported also the formation of two fragment anions, (C_3_H_2_NO)^−^ and NCO^–^, associated with the cleavage of the uracil ring.^26^ We observed the latter anion also in our recent study with BrSdU,^38^ however, with a reduced relative intensity compared to that of brominated nucleoside BrdU.^39^ This quenching of NCO^–^ formation in halogenated thio-2′-deoxyuracils/uridines like ISdU may be explained by the simple argument that one site of formation is not available due to the presence of the sulfur atom. Surprisingly, the thiocyanate SCN^–^ is not observed presently, though the neutral thiocyanate radical represents a pseudohalogen like NCO. Apart from that, the DEA process in ISdU and IU^26^ obviously leads to different outcomes in terms of the abundance of reaction products; also, resonance formation (i.e., initial formation of the temporary negative ion) at low electron energies shows some differences. The fragment anions observed in ref (26) for IU showed, besides the threshold peak at 0 eV, another peak near 1.3 eV, which can be associated with a common TNI (a π* resonance in this case) for fragment anion formation at this energy. In this study, we observe such a common TNI state at a much lower electron energy (∼0.4 eV). In comparison, several fragment anions formed upon DEA to 5-bromo-4-thiouracil (BrSU) showed a resonance feature near 0.5 eV, which may lead to the conclusion that the kind of halogen atom attached has little influence on the energy of this resonance. By means of electron transmission spectroscopy, Scheer et al. investigated low-lying resonances for halouracils 5-XU (X = Cl, Br, or F) and the native uracil nucleobase,^47^ and indeed, their spectra indicated a small shift of only <0.3 eV for the second and third π* resonances if uracil is halogenated at the C5 position. This tendency was also supported by resonance scattering calculations.^48^ In contrast, the resonance energy of the second π* resonance seems to be more strongly affected by the replacement of the oxygen with the sulfur atom. Varella and co-workers reported a resonance energy of 0.56 eV for the second π* resonance in 2-thiouracil,^49^ i.e., red-shifted by ∼1 eV compared to the native uracil.^50^ This red shift was explained by the greater electron affinity of sulfur compared to the oxygen atom.^49^ Thus, we may tentatively ascribe the peak observed near 0.4 eV in the present DEA yields to the initial formation of the π* resonance.

In this work, we found that low-energy electrons with electron energies near 0 eV effectively decompose the ISdU molecule upon electron attachment. No parent anion could be observed in the experiment, while the by far most abundant reaction channel leads to the formation of the I^–^ anion and the SdU^•^ radical, which may also be essential in terms of acting as a potential radiosensitizer. The calculations predict a modest exothermicity for this channel (−0.38 eV), which is almost isoenergetic to the electron attachment-induced cleavage of the glycosidic bond. As the calculations indicate, a higher transition state may limit the release of the anionic nucleobase moiety. Another reason could be less efficient coupling of the π* resonance with the σ*_C–N_ state than with the σ*_C–I_ state.

The electron attachment properties of ISdU observed in this work are in striking contrast to those of 4-thiouracil with Br added at the C5 position, because for the latter compound Br^–^ contributes <10% to the overall fragment ion yield.^38^ The reduced rate of release was explained by competing intramolecular proton transfer reactions favoring anionic products other than Br^–^. We note that for fluorinated uracil this competition was shown to be enhanced,^42,51^ resulting from the high proton affinity of F^–^.^52^ It is important to conclude that for the studied 5-X-4-thio-2′-deoxyuridines/uracils (X = I or Br) the overall DEA tendencies observed here in the gas phase are reflected in the solution phase. The same conclusion seems to apply to halouracils.^26,53^ Thus, DEA studies with such halogen-modified molecules may allow first useful predictions about their potential, though they cannot fully replace radiolysis studies.^54^

To introduce ISdU into the clinic, a huge amount of further work is necessary. Namely, animal studies must be carried out before any clinical tests. Therefore, the rapid dissemination of our results should quickly induce in vivo tests. On the contrary, the cellular mechanism of radiosensitization suggested by our studies should help in the selection of a drug administration scheme.

## Supporting Information Available

Details of the experimental and computational methods, *xyz* geometries of all stationary points (minima and transition states), and raw experimental data (ZIP)
Transparent Peer Review report available (PDF)
