# Supplementary material for: Dissociative Electron Attachment to 5-Iodo-4-thio-2′-deoxyuridine: A Potential Radiosensitizer of Hypoxic Cells
Source: J Phys Chem Lett. 2023 Sep 28;14(40):8948–55. doi: 10.1021/acs.jpclett.3c02219 (PMC10578351; doi:10.1021/acs.jpclett.3c02219)
Supplement: Supplementary file 1 — jz3c02219_si_001.zip [file jz3c02219_si_001.zip › ISdU_SI.docx]

**Supplementary Information**

Dissociative Electron Attachment to 5-Iodo-4-thio-2’-deoxyuridine - a Potential Radiosensitizer of Hypoxic Cells

*Muhammad Saqib,^a,b^ Eugene Arthur-Baidoo,^a,b^ Farhad Izadi,^a,b^ Adrian Szczyrba,^c^ Magdalena Datta,^c^ Sebastian Demkowicz,^d^ Janusz Rak,^c*^ Stephan Denifl,^a,b *^*

^a^ Institut für Ionenphysik und Angewandte Physik, Universität Innsbruck, Technikerstraße 25, A-6020 Innsbruck, Austria.

^b^ Center for Molecular Biosciences Innsbruck, Universität Innsbruck, Technikerstraße 25, A-6020 Innsbruck, Austria

^c^ Laboratory of Biological Sensitizers, Department of Physical Chemistry, Faculty of Chemistry, University of Gdańsk, Wita Stwosza 63, 80-308 Gdańsk, Poland

^d^ Department of Organic Chemistry, Faculty of Chemistry, Gdańsk University of Technology, Narutowicza 11/12, 80-233 Gdańsk, Poland

*Correspondence: [janusz.rak@ug.edu.pl](mailto:janusz.rak@ug.edu.pl); Stephan.Denifl@uibk.ac.at

**S.1. Experimental Methods**

The experimental setup used for the DEA study with ISdU in the gas phase has been described in detail in ref.1. In brief, the system consisted of a crossed electron-molecular beam apparatus coupled with a quadrupole mass spectrometer. The molecular effusive beam in the gas phase was produced by the evaporation of the solid sample in a resistively heated copper oven inside the vacuum chamber. The ISdU sample was synthesized according to the previously reported procedure.^2^ The experiment was performed at chamber pressure and sample temperature of 2 × 10^-7^ mbar and 383 K, respectively. The sample vapor entered the interaction chamber of the hemispherical electron monochromator (HEM) through a capillary of 1 mm diameter where it perpendicularly intersected with a monochromatized electron beam. In the present experiment, the HEM was tuned to an energy resolution of about 130 meV at Full-Width Half-Maximum (FWHM) with an electron current of about 30 nA. The anions formed upon electron attachment to ISdU were extracted from the interaction region by a weak electrostatic field into a quadrupole mass filter where they were mass-selected and further detected by a channeltron electron multiplier in single pulse counting mode. For each anion detected, the ion yield was recorded as a function of the electron energy. Prior to the measurements of negative ions, the temperature dependence of the electron impact ionization mass spectrum at 70 eV was measured. This measurement aimed to ensure that no significant thermal decomposition occurred till the sublimation temperature chosen for the negative ion measurements. The electron energy scale and energy resolution were determined by measuring the well-known ion yield for the formation Cl^–^/CCl_4_ at 0 eV.^3^ The remaining electrons, which crossed the interaction region, were collected by a Faraday plate and monitored using a picoamperemeter.

**S.2. Computational Methods**

From a chemistry point of view, no thermodynamic equilibrium of the reaction mixture with the environment can exist due to the single collision regime of the present CEMB experiment at very low pressure (10^-7^ mbar). Thus, according to the energy conservation rule, the thermodynamic threshold, i.e. the excess electron energy which initiates the DEA process, should be calculated as the electronic energy difference (ΔE) between the anionic product and neutral substrate corrected for zero point energy (ZPE). The redistribution of vibrational energy after electron attachment occurs within the time of molecular vibrations, 10^-13^-10^-12^ s, which is much shorter than the time of the CEMB experiment itself. Therefore, calculating the respective thresholds, one should correct the electronic energy difference between the product and substrate for the differences in zero point energies (ΔE(ZPE)).

The energy difference, ΔE(ZPE), determines the thermodynamic threshold if there is no transition state on the way from the substrate to the product which is the case, for instance, for homolytic bond dissociation. However, if a transition state exists, its electronic energy may be larger than that of the neutral substrate. Thus, even for the exothermic DEA process (a negative ΔE(ZPE)) the excess electron energy may be bigger than zero eV. Similarly, for the endothermic processes, the occurrence of a transition state will make the threshold larger than ΔE(ZPE). Hence, to trigger an electron-attachment induced reaction for which a bottle-neck step proceeds via a transition state, the excess electron has to possess kinetic energy equal to the difference between the energy of the substrate and that of the transition state. Therefore, transition states have to be identified and characterized in order to properly estimate the experimental thresholds.

The geometries of all species involved in the studied fragmentations were fully optimized at the M06-2X^4^ level with the DGDZVP++^5,6^ basis set and the harmonic frequencies were calculated using the Hessian matrix of second derivatives of total energy in respect to nuclear coordinates. All such obtained geometries were geometrically stable, which was confirmed by the analysis of harmonic frequencies (all force constants were positive for minima, while all but one were negative for the first-order transition states). The initial geometries of transition states were identified using the QST2 variant of the Synchronous Transit-Guided Quasi-Newton method.^7^ Such obtained geometries were refined using gradient minimization followed by frequency calculations. Finally, the intrinsic reaction coordinate (IRC)^8,9^ procedure was used to verify that the obtained transition state connects the proper minima. All calculations were performed with the Gaussian 16 suite of programs.^10^

**S.3. XYZ geometries of stationary points**

**ISdU**

Total Electronic Energy: -8078.164227

C 0.459550 0.931794 0.473189

N 0.266157 0.048048 1.506433

C 1.286984 -0.790255 1.940441

N 2.461399 -0.653397 1.222112

C 2.757644 0.205976 0.173470

C 1.629077 1.048772 -0.195197

C -1.024782 -0.003672 2.194271

C -1.322123 1.227418 3.050710

C -2.844247 1.238255 3.051614

C -3.173931 0.751645 1.633666

O -2.049355 -0.041039 1.211087

O -3.251258 0.309558 4.043421

C -3.360440 1.877999 0.627799

O -3.478555 1.374764 -0.684062

O 1.174985 -1.568023 2.867144

S 4.250291 0.199905 -0.512294

I 1.785107 2.426853 -1.755981

H -1.022029 -0.920696 2.783744

H -3.260667 2.234180 3.246342

H -4.054302 0.100017 1.625706

H -4.215050 0.217827 4.027018

H -4.281846 2.425065 0.845005

H -2.521574 2.588744 0.704978

H -2.774163 0.719368 -0.814533

H 3.214172 -1.263936 1.532952

H -0.411061 1.524914 0.213550

H -0.926659 2.128638 2.571406

H -0.913678 1.143096 4.058118

**ISdU^•─^**

Total Electronic Energy: -8078.213490

C 0.000347 0.000078 -0.000164

N 0.000135 0.000133 1.422058

C 1.191550 -0.000001 2.117504

N 2.290174 0.336516 1.374362

C 2.288812 0.855216 0.040556

C 1.115470 0.527742 -0.641732

C -1.197515 -0.422676 2.164169

C -1.475678 0.482098 3.387908

C -2.879562 1.029319 3.137316

C -3.009238 0.903825 1.620283

O -2.327353 -0.312385 1.321162

O -3.808634 0.195078 3.828530

C -2.416564 2.107716 0.886248

O -2.510004 1.970188 -0.514441

O 1.250066 -0.274363 3.320413

S 3.683572 1.675747 -0.474822

I 0.950353 0.874419 -2.722820

H -1.098051 -1.476852 2.439077

H -2.987333 2.068343 3.474447

H -4.044129 0.765622 1.285624

H -4.687912 0.337928 3.450807

H -2.994697 2.997557 1.161317

H -1.375930 2.269291 1.197371

H -1.739727 1.450080 -0.806196

H 3.147841 0.466735 1.898639

H -0.792287 -0.552745 -0.488664

H -0.738435 1.283494 3.440406

H -1.435626 -0.076286 4.322751

**TS_253**

Total Electronic Energy: -8078.171124

O -0.002565 0.007823 0.001630

C 0.000833 -0.004573 1.381548

C 1.422263 -0.013480 1.883144

C 2.209004 0.450695 0.660178

C 1.343731 -0.078191 -0.485380

O 2.258056 1.876948 0.683250

C 1.704921 -1.500488 -0.895453

O 0.950181 -1.915651 -2.014305

N -1.013929 -1.742977 1.822754

C -1.259365 -2.508523 0.736335

C -0.639197 -3.737658 0.473013

C 0.262880 -4.296839 1.393720

N 0.411123 -3.493275 2.528364

C -0.217732 -2.291833 2.815197

O -0.007558 -1.729719 3.891086

S 1.146721 -5.738892 1.307769

I -1.201744 -4.799726 -1.254077

H -0.745363 0.648446 1.817038

H 3.225868 0.039676 0.626766

H 1.393358 0.558617 -1.377736

H 2.481844 2.187619 -0.205267

H 2.758297 -1.521842 -1.196935

H 1.578696 -2.191543 -0.049999

H 0.119343 -2.300714 -1.691456

H 0.984090 -3.879469 3.273165

H -2.002713 -2.119606 0.044045

H 1.750831 -1.021551 2.173085

H 1.571741 0.640567 2.742859

**complex_253**

Total Electronic Energy: -8078.196959

O 0.254483 2.886001 -0.504302

C 0.262933 2.490209 0.818436

C 1.656295 2.506092 1.379528

C 2.459680 3.178772 0.263984

C 1.604576 2.903241 -0.977211

O 2.537365 4.574029 0.558512

C 1.948300 1.584587 -1.659632

O 1.148804 1.387028 -2.811636

N -2.098813 0.450345 2.397417

C -1.980977 -0.069539 1.187703

C -0.833002 -0.656555 0.646764

C 0.335988 -0.740668 1.432840

N 0.154619 -0.214430 2.690716

C -0.991020 0.415995 3.207305

O -0.933765 0.888951 4.341457

S 1.851321 -1.368911 1.012714

I -0.859287 -1.322183 -1.339717

H -0.645907 2.725401 1.358802

H 3.467926 2.759041 0.161187

H 1.667276 3.710278 -1.717517

H 2.790463 5.041930 -0.249645

H 2.989699 1.616297 -1.997385

H 1.844676 0.748561 -0.951532

H 0.297027 1.030593 -2.510171

H 0.956293 -0.237323 3.314727

H -2.874236 -0.017123 0.564287

H 2.034798 1.489803 1.556489

H 1.741170 3.073622 2.310635

**(ISdU-deoxyribose)^–^**

Total Electronic Energy: -7656.758487

C -0.000000 -0.000000 0.000000

N -0.000000 -0.000000 1.323657

C 1.221242 -0.000000 1.948921

N 2.357668 -0.000131 1.122259

C 2.411477 -0.000281 -0.254637

C 1.121146 -0.000043 -0.833588

O 1.387242 0.000106 3.168829

S 3.914075 0.000093 -1.025873

I 0.855560 0.000185 -2.913725

H 3.249313 -0.000048 1.609026

H -0.985124 0.000120 -0.468214

**deoxyribose^•^**

Total Electronic Energy: -421.411035

O 0.000000 0.000000 -0.000000

C 0.000000 0.000000 1.374471

C 1.400220 0.000000 1.905218

C 2.162842 0.628094 0.737589

C 1.336576 0.176238 -0.477106

O 2.126318 2.034868 0.920759

C 1.844622 -1.125535 -1.074987

O 1.099394 -1.381693 -2.251316

H -0.853007 -0.497441 1.820304

H 1.780708 -1.012899 2.108239

H 3.199987 0.276548 0.675332

H 1.310251 0.938311 -1.263727

H 2.463659 2.468355 0.123765

H 2.914416 -1.021715 -1.305982

H 1.719975 -1.930368 -0.337547

H 1.244957 -2.295829 -2.529005

H 1.523259 0.597494 2.811298

**TS_127**

Total Electronic Energy: -8077.999888

O 2.546224 -0.828750 -1.084614

C 2.554743 0.541421 -0.754516

C 3.167375 0.657598 0.661198

C 3.628426 -0.761982 0.978703

C 2.707416 -1.609729 0.099949

N 1.175245 1.061425 -0.801394

C 0.053136 0.233202 -1.002217

C -1.142632 0.607707 -0.496368

C -1.315666 1.884599 0.082007

N -0.313250 2.784294 -0.360515

C 1.006581 2.394601 -0.530334

O 1.953457 3.178714 -0.436327

S -2.497861 2.442006 1.131496

I -2.692410 -1.184975 -0.093448

O 4.995501 -0.871522 0.584793

C 1.387623 -1.950445 0.790680

O 0.617198 -2.765008 -0.065706

H 3.144140 1.072871 -1.503152

H 3.523075 -1.008484 2.042444

H 3.175855 -2.548889 -0.217817

H 5.215434 -1.810110 0.501223

H 1.624396 -2.494376 1.718083

H 0.847410 -1.035795 1.067330

H -0.315064 -2.474616 -0.037830

H -0.415066 3.744861 -0.044298

H 0.237431 -0.676239 -1.564243

H 2.409309 0.994846 1.371789

H 4.001576 1.358158 0.688871

**complex_127**

Total Electronic Energy: -8078.213980

C -0.033509 -0.112429 -0.062286

N 0.029844 -0.067141 1.306602

C 1.250972 -0.180285 1.973676

C 2.420028 -0.397384 1.338870

C 2.434099 -0.467375 -0.091573

N 1.189166 -0.242160 -0.686814

C -1.270109 -0.146805 2.015360

O -1.061987 -0.047959 3.401194

C -1.268210 -1.321190 4.021920

C -2.337734 -1.986139 3.155842

C -1.974582 -1.498043 1.757818

O -3.646170 -1.502101 3.452000

C 0.008558 -2.151873 4.136518

O 0.906838 -1.470669 4.978529

I 3.986199 -1.747726 3.206474

S 3.743733 -0.761813 -1.073269

O -1.094241 -0.055960 -0.686450

H -1.872068 0.701885 1.688600

H -2.311431 -3.079583 3.237551

H -1.622538 -1.106344 5.036779

H -3.778086 -1.539946 4.409921

H -0.261954 -3.130138 4.563700

H 0.448463 -2.335239 3.145473

H 1.823519 -1.618858 4.658972

H 1.147640 -0.286488 -1.702179

H 1.171509 -0.090028 3.052140

H -1.288532 -2.192627 1.266809

H -2.858337 -1.383736 1.131398

**I^−^**

Total Electronic Energy: -6920.358373

I 0.000000 0.000000 0.000000

**SdU^•^**

Total Electronic Energy: -1157.608254

C 1.098752 0.802074 -0.666409

H 1.088107 1.553280 -1.457525

C 1.783728 1.306510 0.619073

C 3.087222 0.511872 0.658562

H 3.419927 0.307778 1.683879

O 4.055327 1.269362 -0.047439

C 2.706309 -0.766937 -0.091184

H 3.552469 -1.229341 -0.607837

C 2.054114 -1.799576 0.817609

O 1.645471 -2.885098 0.002497

O 1.810987 -0.319470 -1.112481

N -0.318894 0.404534 -0.427631

C -1.174705 1.426097 -0.052492

O -0.792893 2.575460 0.097182

N -2.493905 1.047396 0.149935

C -3.043090 -0.220290 0.003320

S -4.636945 -0.535548 0.285577

C -2.067463 -1.165652 -0.415098

C -0.777912 -0.884456 -0.642922

H 4.869829 0.753085 -0.134541

H 2.786734 -2.120583 1.569780

H 1.193770 -1.356365 1.341449

H 1.229752 -3.560906 0.556488

H -3.118945 1.802330 0.427244

H -0.032292 -1.599616 -0.980191

H 1.163220 1.098044 1.493404

H 1.975274 2.377659 0.581670

**TS_126b**

Total Electronic Energy: -8078.161005

O -3.175937 0.234596 -1.104824

C -2.736839 -1.084650 -1.079833

C -3.423927 -1.774245 0.112996

C -4.375289 -0.683402 0.633848

C -3.644069 0.589403 0.208300

I -0.482904 -1.155793 -0.898036

C 2.032658 -0.565353 -0.134649

C 2.227975 0.227109 0.991924

N 3.553828 0.267665 1.359280

C 4.636911 -0.367980 0.716895

N 4.357796 -1.104291 -0.407785

C 3.086519 -1.172832 -0.795044

S 1.083901 1.092058 1.917775

O 5.760587 -0.220719 1.198519

O -5.661488 -0.765377 0.028266

C -2.532921 0.992355 1.175958

O -1.786306 2.051375 0.635898

H -2.909858 -1.535228 -2.054790

H -4.538926 -0.746596 1.710744

H -4.326288 1.430540 0.063101

H -5.556042 -0.497207 -0.898659

H -3.028238 1.302224 2.109719

H -1.885399 0.140147 1.417456

H -0.836723 1.842177 0.768640

H 3.795664 0.823423 2.175856

H 2.895483 -1.762289 -1.693241

H -2.704146 -2.066058 0.879886

H -3.982686 -2.660643 -0.195020

**complex_126b**

Total Electronic Energy: -8078.164156

C 0.155953 0.689694 0.078753

C 0.125422 0.782394 1.458035

C 1.100313 0.197475 2.253032

N 2.056355 -0.450759 1.508150

C 2.109849 -0.547499 0.101003

N 1.104324 0.056484 -0.611352

S 1.210242 0.212902 3.956006

O 3.051302 -1.166219 -0.398049

I -2.815361 -0.399227 2.634456

C -4.119631 -0.522438 4.418701

O -3.963244 0.595999 5.232519

C -3.128194 0.243914 6.351022

C -3.633965 -1.158304 6.686216

C -3.812925 -1.762428 5.283615

O -4.862501 -1.105605 7.403999

C -1.635711 0.279252 6.043507

O -1.282902 1.552779 5.571647

H -5.117102 -0.500927 3.987003

H -2.940460 -1.730777 7.303882

H -3.362512 0.968697 7.134499

H -5.405737 -0.416849 6.988469

H -1.113690 0.042905 6.985247

H -1.360374 -0.495821 5.316944

H -0.564041 1.423034 4.914656

H 2.815665 -0.907660 2.006714

H -0.631264 1.154008 -0.516887

H -2.904140 -2.269295 4.956755

H -4.637461 -2.478005 5.269679

**SU(N3-H)^•−^**

Total Electronic Energy: -736.419160

C -0.040554 0.034292 0.020058

N 0.005375 0.095032 1.326363

C 1.293419 -0.006610 1.945760

N 2.454416 0.040046 1.201310

C 2.369964 0.003161 -0.119719

C 1.105092 -0.071930 -0.790819

O 1.312936 -0.118680 3.173245

S 3.752880 0.101450 -1.149492

H -1.030721 0.078894 -0.440948

H 1.053892 -0.155194 -1.869314

**(deoxyribose + I)**

Total Electronic Energy: -7341.736928

C -0.000241 0.000479 -0.000492

O -0.000016 0.001378 1.429807

C 1.309034 0.000601 1.856369

C 2.061868 -0.906967 0.889787

C 1.294124 -0.734513 -0.426823

I 2.163693 2.064641 1.847291

O 2.088665 0.019702 -1.325275

C -1.265763 -0.660247 -0.496324

O -2.358503 0.188019 -0.206102

H 1.348727 -0.266838 2.907758

H 1.964146 -1.933566 1.255945

H 1.056384 -1.710831 -0.868615

H 0.042506 1.041128 -0.349169

H 1.586651 0.182845 -2.137243

H -1.168615 -0.821469 -1.580913

H -1.367935 -1.638242 -0.006778

H -3.184285 -0.295994 -0.341442

H 3.116707 -0.667160 0.766867

**TS1_126a**

Total Electronic Energy: -8078.146102

O 2.617734 -1.373608 -0.385912

C 1.991073 -0.230378 -0.853178

C 3.053088 0.780437 -1.250527

C 4.362173 0.022684 -0.958553

C 3.903392 -0.966354 0.113506

O 4.857573 -0.660080 -2.108942

C 3.802526 -0.300291 1.492376

O 2.946133 -1.006799 2.360176

N 0.847708 0.242630 0.750361

C -0.284352 -0.488616 0.652302

C -1.490672 0.072652 0.221080

C -1.511687 1.454943 0.034806

N -0.467640 2.233676 0.312118

C 0.708106 1.661146 0.715487

O 1.698162 2.343383 1.021901

S -2.978410 2.276353 -0.562629

I -3.167507 -1.162719 -0.094108

H 1.166196 -0.467614 -1.522301

H 5.170093 0.673966 -0.619619

H 4.517522 -1.869327 0.149573

H 4.179380 -1.310085 -2.353760

H 4.804731 -0.292395 1.936086

H 3.458707 0.737469 1.388583

H 2.051555 -0.736032 2.064713

H -2.400113 3.484124 -0.473956

H -0.195251 -1.557284 0.839677

H 2.964639 1.691933 -0.649528

H 2.995811 1.053031 -2.308584

**complex1_126a**

Total Electronic Energy: -8078.175271

O 1.994575 0.922652 -1.900509

C 2.137460 1.064800 -0.538935

C 3.568804 0.883249 -0.123134

C 4.286177 0.963092 -1.482135

C 3.226030 0.391799 -2.427848

O 4.615888 2.308809 -1.824770

C 3.239587 -1.137263 -2.444555

O 2.124402 -1.680472 -3.112053

N 0.609190 -1.711980 -0.695789

C -0.494013 -0.992521 -0.715085

C -0.994256 -0.309375 0.394959

C -0.250390 -0.458990 1.578636

N 0.857864 -1.153203 1.644301

C 1.350819 -1.755354 0.486695

O 2.452265 -2.321460 0.507703

S -0.805505 0.328822 3.084456

I -2.752076 0.829657 0.250751

H 1.361951 1.644160 -0.054144

H 5.223002 0.403931 -1.512889

H 3.320757 0.788396 -3.442237

H 3.780887 2.803951 -1.831747

H 4.147643 -1.444421 -2.980898

H 3.297843 -1.530412 -1.420886

H 1.463872 -1.845654 -2.400019

H 0.244818 -0.140044 3.775901

H -1.025349 -0.935263 -1.665720

H 3.721244 -0.097618 0.350519

H 3.933928 1.657081 0.558374

**TS2_126a**

Total Electronic Energy: -8078.112062

O 2.964092 0.044416 1.161524

C 2.572543 -1.055266 0.371116

C 3.274185 -0.897608 -0.992456

C 4.215083 0.293711 -0.759828

C 3.450368 1.083637 0.301593

O 5.486303 -0.117958 -0.269126

C 2.340941 1.965126 -0.271882

O 1.587633 2.577086 0.749948

I 0.361959 -1.131791 0.197713

C -2.426896 -0.614947 -0.381704

C -2.798297 0.730988 -0.266577

N -4.010245 1.118325 0.039458

C -5.002520 0.125345 0.155962

N -4.745909 -1.188255 -0.272268

C -3.500408 -1.500116 -0.559404

S -1.546640 1.992290 -0.501488

O -6.112210 0.426642 0.595256

H -2.388330 3.001280 -0.224903

H -3.320454 -2.518896 -0.910422

H 2.833109 -1.965342 0.908805

H 2.558672 -0.677863 -1.787852

H 3.838074 -1.789929 -1.271497

H 5.349462 -0.445148 0.634633

H 4.408653 0.864733 -1.669726

H 4.114206 1.698834 0.914510

H 2.822134 2.760965 -0.852767

H 1.693941 1.399367 -0.954276

H 0.789513 2.043083 0.906426

**complex2_126a**

Total Electronic Energy: -8078.118870

C 0.169307 -0.019739 -0.211472

C 0.107925 -0.293340 1.153551

C 1.292211 -0.148389 1.867546

N 2.363562 0.416378 1.367123

C 2.323807 0.844963 0.026458

N 1.215608 0.547920 -0.780077

S 1.375756 -0.742353 3.561327

O 3.284883 1.461346 -0.440750

I -2.259778 0.314142 3.117295

C -3.498088 0.372749 4.945776

O -2.830271 1.052908 5.974918

C -2.309742 0.093042 6.906847

C -3.430744 -0.944349 6.958536

C -3.833852 -1.034827 5.479059

O -4.504369 -0.496485 7.778122

C -0.957703 -0.487939 6.495977

O 0.007377 0.529205 6.363269

H 2.653178 -0.360020 3.719467

H -0.675597 -0.253123 -0.863033

H -4.363191 0.963747 4.652947

H -3.258827 -1.807210 4.965151

H -4.895654 -1.267470 5.377629

H -4.653160 0.439772 7.569109

H -3.113028 -1.904676 7.368255

H -2.196854 0.624585 7.855093

H -0.641498 -1.168853 7.297107

H -1.041081 -1.077101 5.574182

H 0.218756 0.631944 5.418104

**TS3_126a**

Total Electronic Energy: -8078.087071

C 0.064351 0.132779 0.118976

C 0.047812 -0.095255 1.630003

O 1.449293 -0.147891 1.937760

C 2.112265 -0.845824 0.920265

C 1.196534 -0.807335 -0.321603

C -0.722849 -1.342891 2.054371

O -0.630144 -1.506528 3.447843

I 2.578351 -2.906831 1.550758

O 0.351664 1.492636 -0.188892

C 2.012561 -5.819725 3.067276

C 1.196866 -5.405080 4.185123

N 1.487404 -5.463543 5.445197

C 2.728151 -6.070457 5.723484

N 3.403466 -6.841544 4.744198

C 3.007891 -6.744619 3.496051

S -0.323063 -4.895874 3.429242

O 3.217383 -5.981928 6.844719

H 3.089020 -0.389940 0.775695

H -0.886615 -0.096572 -0.364613

H -0.347186 0.768082 2.171560

H 1.060932 1.776339 0.410244

H -1.772423 -1.187906 1.762011

H -0.357537 -2.230802 1.523877

H -0.338316 -2.418513 3.636264

H 0.708333 -5.294406 2.330728

H 3.539361 -7.367609 2.773188

H 0.808007 -1.797528 -0.565726

H 1.713956 -0.410972 -1.197536

**complex3_126a**

Total Electronic Energy: -8078.195556

C -1.683338 2.447892 -1.560113

C -1.126116 2.915237 -0.216267

O 0.290581 3.006670 -0.492712

C 0.653408 2.010261 -1.386286

C -0.631771 1.408439 -1.970799

C -1.480604 1.952806 0.907321

O -0.774738 2.264360 2.083865

I 1.950747 0.479154 -0.422862

O -1.773788 3.526071 -2.490667

C 0.207664 -3.210425 -0.317499

C -0.824881 -2.238234 -0.465823

N -1.087227 -1.351340 0.491665

C -0.326657 -1.365024 1.634620

N 0.619067 -2.390916 1.893448

C 0.869572 -3.235667 0.925356

S -1.705802 -2.269182 -1.946064

O -0.442412 -0.477202 2.496621

H 1.344957 2.422383 -2.118194

H -2.681416 2.014810 -1.485197

H -1.435046 3.933936 0.033548

H -0.965704 4.054087 -2.394014

H -2.567573 2.049617 1.065078

H -1.295545 0.915494 0.607203

H -0.517893 1.395683 2.456246

H 0.436594 -3.903507 -1.117049

H 1.624488 -3.999195 1.123562

H -0.870205 0.437904 -1.527812

H -0.573250 1.294332 -3.054696

**SU(C5-H)^•−^**

Total Electronic Energy: -736.403008

C -0.000000 -0.000000 0.000000

N -0.000000 -0.000000 1.338935

C 1.215871 0.000000 1.967595

N 2.370139 0.000088 1.153228

C 2.413374 0.000117 -0.222537

C 1.136275 0.000070 -0.777968

O 1.377367 0.000106 3.191801

S 3.893588 0.000174 -1.056595

H 3.257121 0.000133 1.650124

H -0.985679 -0.000034 -0.467803

**S.4. References**

(1) Saqib, M.; Izadi, F.; Isierhienrhien, L. U.; Ončák, M.; Denifl, S. Decomposition of Triazole and 3-Nitrotriazole upon Low-Energy Electron Attachment. *Phys. Chem. Chem. Phys.* **2023**, *25*, 13892-13901.

(2) Makurat, S.; Spisz, P.; Kozak, W.; Rak, J.; Zdrowowicz, M. 5-Iodo-4-Thio-2′-Deoxyuridine as a Sensitizer of X-Ray Induced Cancer Cell Killing. *Int. J. Mol. Sci.* **2019**, *20*(6), 1308.

(3) Klar, D.; Ruf, M. W.; Hotop, H. Dissociative Electron Attachment to CCl_4_ Molecules at Low Electron Energies with MeV Resolution. *Int. J. Mass. Spectrom.* **2001**, *205*(1–3), 93-110.

(4) Zhao, Y.; Truhlar, D. G. The M06 Suite of Density Functionals for Main Group Thermochemistry, Thermochemical Kinetics, Noncovalent Interactions, Excited States, and Transition Elements: Two New Functionals and Systematic Testing of Four M06-Class Functionals and 12 Other Functionals. *Theor. Chem. Acc.* **2008**, *120*(1–3), 215-247.

(5) Godbout, N.; Salahub, D. R.; Andzelm, J.; Wimmer, E. Optimization of Gaussian-Type Basis Sets for Local Spin Density Functional Calculations. Part I. Boron through Neon, Optimization Technique and Validation. *Can. J. Chem.* **1992**, *70*(2), 560-571.

(6) Sosa, C.; Andzelm, J.; Elkin, B. C.; Wimmer, E.; Dobbs, K. D.; Dixon, D. A. A Local Density Functional Study of the Structure and Vibrational Frequencies of Molecular Transition-Metal Compounds. *J. Phys. Chem.* **1992**, *96*(16), 6630-6636.

(7) Peng, C.; Bernhard Schlegel, H. Combining Synchronous Transit and Quasi‐Newton Methods to Find Transition States. *Isr. J. Chem.* **1993**, *33*(4), 449-454.

(8) Fukui, K. The Path of Chemical Reactions - The IRC Approach. *Acc. Chem. Res.* **1981**, *14*(12), 363-368.

(9) Hratchian, H. P.; Schlegel, H. B. Finding Minima, Transition States, and Following Reaction Pathways on Ab Initio Potential Energy Surfaces. In *Theory and Applications of Computational Chemistry: The First Forty Years*. **2005**, 195-249.

(10) Frisch, M. J.; Trucks, G. W.; Schlegel, H. B.; Scuseria, G. E.; Robb, M. a.; Cheeseman, J. R.; Scalmani, G.; Barone, V.; Petersson, G. a.; Nakatsuji, H.; Li, X.; Caricato, M.; Marenich, a. V.; Bloino, J.; Janesko, B. G.; Gomperts, R.; Mennucci, B.; Hratchian, H. P.; Ortiz, J. V.; Izmaylov, a. F.; Sonnenberg, J. L.; Williams; Ding, F.; Lipparini, F.; Egidi, F.; Goings, J.; Peng, B.; Petrone, A.; Henderson, T.; Ranasinghe, D.; Zakrzewski, V. G.; Gao, J.; Rega, N.; Zheng, G.; Liang, W.; Hada, M.; Ehara, M.; Toyota, K.; Fukuda, R.; Hasegawa, J.; Ishida, M.; Nakajima, T.; Honda, Y.; Kitao, O.; Nakai, H.; Vreven, T.; Throssell, K.; Montgomery Jr., J. a.; Peralta, J. E.; Ogliaro, F.; Bearpark, M. J.; Heyd, J. J.; Brothers, E. N.; Kudin, K. N.; Staroverov, V. N.; Keith, T. a.; Kobayashi, R.; Normand, J.; Raghavachari, K.; Rendell, a. P.; Burant, J. C.; Iyengar, S. S.; Tomasi, J.; Cossi, M.; Millam, J. M.; Klene, M.; Adamo, C.; Cammi, R.; Ochterski, J. W.; Martin, R. L.; Morokuma, K.; Farkas, O.; Foresman, J. B.; Fox, D. J. Gaussian 16. 2016.
