# Supplementary material for: Dissociative Electron Attachment to 5-Iodo-4-thio-2′-deoxyuridine: A Potential Radiosensitizer of Hypoxic Cells
Source: J Phys Chem Lett. 2023 Sep 28;14(40):8948–55. doi: 10.1021/acs.jpclett.3c02219 (PMC10578351; doi:10.1021/acs.jpclett.3c02219)
Supplement: Supplementary file 2 — jz3c02219_si_002.pdf [file jz3c02219_si_002.pdf]

jz-2023-022192.R1

Name: Peer Review Information for "Dissociative Electron Attachment to 5-Iodo-4-thio-2'-deoxyuridine - a Potential Radiosensitizer of Hypoxic Cells"

First Round of Reviewer Comments

Reviewer: 1

Comments to the Author

The authors investigated experimentally dissociative electron attachment (DEA) to 5-iodo-4-thio-2'-deoxyuridine (ISdU), using a molecular beam crossed by a monoenergetic electron beam, both couple to a mass spectrometer for measuring anion yields. The experimental results are supported by calculations of the transition states and threshold energies of the formed anions. In recent work, the potential of ISdU as an effective radiosensitizer was demonstrated in studies involving clonogenic assays and steady state radiolysis. The radiosensitizing properties of 5-iodo-4-thio-2'-deoxyuridine (ISdU) in solution were also recently investigated and the use of the compound as a potential photosensitizer was suggested. In this work, the authors find experimentally that the formation of the halogen anion I- corresponds by far to the predominant process. This implies that the corresponding radical of the DEA process is also produced in equivalent quantities. This highly reactive radical is expected to produce a strong radiosensitization, which corroborates previous studies. In fact, due to DEA bond cleavage, a radical site is formed at the C5 position, which may initiate strand break formation if the molecule is incorporated into a DNA strand. Moreover, this bond rupture occurs near zero eV, i.e., at an energy where the largest quantity of electrons is found, i.e., near the end of the energy degradation process.

I am impressed by this work, but not surprised, since this team of researchers is the best I know, to describe the physico-chemical sensitizing properties of halogenated radiosensitizers. Here, these authors have clearly shown that ISdU could be a very efficient radiosensitizer. Both at the theoretical and experimental level the investigations are well done, the results are sound, and the conclusions are a logical consequence of the results. I have little to comment on such excellent work. I would just like to mention that their sentence in the first paragraph of the paper “If the lifetime of the TNI formed is long enough with respect to the spontaneous emission of the excess electron, the decay of the TNI will lead to fragment anion and neutral(s) formation” appears incomplete. It should read “If the TNI state is dissociative in the Frack-Condon region and its lifetime is long enough with respect to autoionization, the decay of the TNI can lead to fragment anion and neutral(s) formation”. It should be realised, however that such studies do not necessarily imply that the process occurs if the compound is incorporated within DNA, particularly if chemically bound to DNA. The parameters of TNIs are sensitive to phase of condensed matter and even more to chemical bonding. Fortunately, evidence is given in a reference on ISdU sensitization of cells to ionizing radiation. The compound probably incorporates into genomic DNA via the action of enzymes, and as a result radio-sensitization occurs. Also, when incorporated into the DNA of cultured human and mouse cells, ISdU synergistically sensitises these cells to low doses of UVA radiation. DNA-protein crosslinks, DNA strand breaks, nucleobase damage lesions were observed with UV photons. These lesions are potentially lethal DNA lesions (R. Brem et al. J Photochem Photobiol B, 2015 Apr;145:1-10). UVA radiation can ionize cellular media, and the liberated electron could induce a SSB if close to DNA, even with energy near zero eV. According to the present data, the initial electron

capture via a shape resonance mechanism should be enhanced by ISdU incorporation into DNA and promote the creation of a highly reactive radical.

This brings me to the question: why should this work be published rapidly as a letter? Good, promising, reliable results do not necessarily need rapid dissemination. At this level, I don't think the authors are very convincing and should try to improve. In my opinion, the importance of rapid dissemination lies in the very high efficiency of potential radiosensitization, which should encourage further research, including with cancer cells and animals and clinical trials. In other words, much work remains to be done to confirm in practice the potential for cancer or other radiation treatments. Thus, considering the importance of the topic and the amount of work ahead, it is important not to waste time and publish as soon as possible. The present determination of the molecular mechanism of radiosenitization by ISdU appears as another reason for rapid publication. There are several ways different drugs can increase the effects of radiation. One of them is an increase of the initial damage to DNA, as shown in the present article. Other mechanisms, such as lowering the threshold of apoptosis of cancer cells, often rely on biological response, which is slow (minutes to days) compared to the immediate effect of DEA. In other words, the mechanism of radiosensitization must be known to effectively establish appropriate protocols (e.g., to determine when to apply the radiation after injection of a radiosensitizer; T. Tippayamontri et al, Anticancer Research 34(10), 5303-5312 (2014)). I therefore suggest publication in the Journal of Physical Chemistry Letters if the authors can explain the urgency of publication in responding convincingly to my comments.

Reviewer: 2

Comments to the Author

This work is technically outstanding and clearly shows low energy electron induced dehalogenation via a cross molecular beam study backed up DFT calculations. The authors are experts in this field and the work is well done and highly likely to be correct. The search for a radiosensitizer in hypoxic cells is a worthy goal but since the cellular systems are not gas phase it is doubtful that this gas phase work is applicable to hypoxic cells as suggested in the title. Studiies in aqueous systems by pulse radiolysis on similar systems show the dehalgenation chemistry as well.

To summarize the work is an outstanding experimental and theoretical work that should be published in J Phys Chem for example, but the action of the molecule as a radiosensitizer is not investigated and limits the impact of the work.

Author's Response to Peer Review Comments:

Reply to the comments and suggestions of the Referees

We would like to thank the Referees for their high opinion of our paper and valuable comments which helped us improve the current work's quality. Our detailed response and the list of corrections addressing all their remarks are listed below.

Referee #1

1) *"I am impressed by this work, but not surprised, since this team of researchers is the best I know, to describe the physico-chemical sensitizing properties of halogenated radiosensitizers. Here, these authors have clearly shown that ISdU could be a very efficient radiosensitizer. Both at the theoretical and experimental level the investigations are well done, the results are sound, and the conclusions are a logical consequence of the results. I have little to comment on such excellent work."*

We are delighted with such a high opinion of our work. Thank you very much.

2) *"I would just like to mention that their sentence in the first paragraph of the paper "If the lifetime of the TNI formed is long enough with respect to the spontaneous emission of the excess electron, the decay of the TNI will lead to fragment anion and neutral(s) formation" appears incomplete. It should read "If the TNI state is dissociative in the Frack-Condon region and its lifetime is long enough with respect to autoionization, the decay of the TNI can lead to fragment anion and neutral(s) formation".*

This sentence has been reformulated according to the Referee's suggestion and now it reads as follows: "If the TNI state is dissociative in the Frack-Condon region and its lifetime is long enough with respect to autoionization, the decay of the TNI can lead to fragment anion and neutral(s) formation" – see p. 6 l. 7-9 in the revised manuscript.

3) *"It should be realized, however that such studies do not necessarily imply that the process occurs if the compound is incorporated within DNA, particularly if chemically bound to DNA. The parameters of TNIs are sensitive to phase of condensed matter and even more to chemical bonding. Fortunately, evidence is given in a reference on ISdU sensitization of cells to ionizing radiation."*

We fully agree with the Referee's comments. To emphasize the fact that ISdU possesses radiosensitizing properties, which can, however, be doubted taking into account the possible influence of DNA double-helix on TNI formation, we added the below fragment:
"One can argue that since the above findings reflect a situation of the isolated ISdU interacting with the excess electron, they may not hold for ISdU incorporated into DNA, as double helix influences the formation of TNI. It is, however, worth noticing that our previous in vitro studies on ISdU confirm the radiosensitizing properties of the modified nucleoside against breast cancer cells. Thus, also being a part of DNA ISdU seems to be prone to DEA." – see p. 12 l. 17-21 in the revised manuscript.

4) *"ISdU synergistically sensitises these cells to low doses of UVA radiation. DNA-protein crosslinks, DNA strand breaks, nucleobase damage lesions were observed with UV photons. These lesions are potentially lethal DNA lesions (R. Brem et al. J Photochem Photobiol B, 2015 Apr;145:1-10)."*

In the introduction, we added two sentences along with a reference to Brem et al. (the reference indicated by the Referee) showing that ISdU possesses not only radiosensitizing but also photosensitizing properties. The respective fragment reads as follows: "Due to the presence of a sulfur atom in the molecules, they absorb in the UVA region (350 nm) far behind the maximum of DNA absorption (260 nm), and irradiation of DNA labeled with these nucleoside modifications leads to interstrand crosslinks and DNA strand breaks.[34]" – see p. 5, l. 21-24.

5) *"According to the present data, the initial electron capture via a shape resonance mechanism should be enhanced by ISdU incorporation into DNA and promote the creation of a highly reactive radical. This brings me to the question: why should this work be published rapidly as a letter? Good, promising, reliable results do not necessarily need rapid dissemination. At this level, I don't think the authors are very convincing and should try to improve. In my opinion, the importance of rapid dissemination lies in the very high efficiency of potential radiosensitization, which should encourage further research, including with cancer cells and animals and clinical trials. In other words, much work remains to be done to confirm in practice the potential for cancer or other radiation treatments. Thus, considering the importance of the topic and the amount of work ahead, it is important not to waste time and publish as soon as possible."*

Thank you for this comment. In order to justify the necessity of rapid dissemination of our findings, we followed the above-mentioned suggestion of the Referee. Namely, we emphasized in the introduction, and conclusions the fact that *in vivo* studies are still necessary to prove ISdU to be an efficient clinical radiosensitizer. In particular, animal studies must precede clinical trials. Hence, the quicker in vivo studies are carried out, the higher are chances for clinical trials. Therefore, rapid dissemination seems to be one of the conditions for working out an efficient clinical radiosensitizer based on the ISdU molecule. To this end, the following modifications have been introduced: (i) in the introduction part: "…confirming the strong radiosensitizing potential of the studied system. However, in order to introduce ISdU to clinical practice huge amount of work is still needed. In particular, positive animal tests are required to initiate clinical trials. The sooner these in vivo studies are carried out, the better is chance of introducing ISdU into clinics. Therefore, rapid dissemination of our results, assured by the letter form of the current paper, is well justified." – see p. 6, l. 12-16, (ii) and in conclusions: "For introducing ISdU into the clinic a huge amount of further work is necessary. Namely, animal studies have to be carried out before any clinical tests. Therefore, rapid dissemination of the current result should quickly induce in vivo tests." – see p. 18, l. 4-6.

6) *"The present determination of the molecular mechanism of radiosenitization by ISdU appears as another reason for rapid publication. There are several ways different drugs can increase the effects of radiation. One of them is an increase of the initial damage to DNA, as shown in the present article. Other mechanisms, such as lowering the threshold of apoptosis of cancer cells, often rely on biological response, which is slow (minutes to days) compared to the immediate effect of DEA. In other words, the mechanism of radiosensitization must be known to effectively establish appropriate protocols (e.g., to determine when to apply the radiation after injection of a radiosensitizer; T. Tippayamontri et al, Anticancer Research 34(10), 5303-5312 (2014)). I therefore*

suggest publication in the Journal of Physical Chemistry Letters if the authors can explain the urgency of publication in responding convincingly to my comments. "

As suggested, we also underlined the mechanism of radiosensitization as one of the reasons for urgent publication. Indeed, as indicated by the Reviewer, the mechanism constitutes a basis for compound administration. We added two fragments and a citation to the indicated Prof. Sanche's article: (i) to the body of the paper: "This result indicates, thus, the main radiosensitization mechanism of ISdU at the cellular level. Namely, after its enzymatic incorporation into DNA and attaching a solvated electron, the reactive thiouracil-5-yl radical is formed which induces DNA strand break. Hence, this finding suggests that ISdU should be administrated well before the actual irradiation during radiotherapy. A similar conclusion was drawn from radiotherapy studies on oxaliplatin in mice xenografts.[44] Specifically, it was demonstrated that the highest radiosensitizing effect occurs when the oxaliplatin concentration in DNA reaches the maximum, i.e. after 48 h of drug administration. Hence, the mechanistic information is crucial for further in vivo studies and justifies, similarly to the necessity of doing animal experiments, a rapid publication of the current paper." – see p. 12, l. 22-25, and p. 13, l. 1-5, (ii) to the conclusions: "On the other hand, the cellular mechanism of radiosensitization suggested by our studies should help in selecting a drug administration scheme." – see p. 18, l. 7-8.

Referee #2

1) *Recommendation: Reconsider as an article in The Journal of Physical Chemistry A/B/C.*

   See our reasoning in response to points 5 and 6 of the Reviewer #1 comments.

2) *"This work is technically outstanding and clearly shows low energy electron induced dehalogenation via a cross molecular beam study backed up DFT calculations. The authors are experts in this field and the work is well done and highly likely to be correct."*

   Thank you so much for such a high opinion of our work.

3) *"The search for a radiosensitizer in hypoxic cells is a worthy goal but since the cellular systems are not gas phase it is doubtful that this gas phase work is applicable to hypoxic cells as suggested in the title.*

   The Referee is right saying that gas phase results are not necessarily related to processes occurring in a solution. However, as indicated by our studies the radiosensitizing action of ISdU is related to electron attachment to the nucleoside. Therefore, one can safely state that the process has to occur under hypoxia as far as electron attachment is concerned. Indeed, under normoxia ($[O_2] = 2.5 \times 10^{-3}$ M) oxygen competes for solvated electrons with ISdU molecules and transforms them into the O_2^- radicals, unreactive toward DNA. Moreover, the proposed mechanism (suggested also by refs. 32 and 33)

does not require oxygen for radiosensitizing action. To emphasize this we added the following sentence to the revised manuscript: "Finally, the above description clearly demonstrates that ISdU can radiosensitize cells only under hypoxia. Indeed, oxygen, of a relatively high concentration under normoxia (1.5 x 10^{-3} M),[45] competes with ISdU for solvated electrons forming the O_2^{-} radical, unreactive toward DNA." – see p. 13, l. 6-9.

4) *To summarize the work is an outstanding experimental and theoretical work… but the action of the molecule as a radiosensitizer is not investigated and limits the impact of the work."*

We have already published (and cited the respective papers in the manuscript (see p. 5, l. 20 and p. 6, l. 3)) the outcome of our radiolytic (in aqueous solution; ref. 32) and *in vitro* (ref. 33) studies which demonstrate the radiosensitizing potential of ISdU. The current results confirm the radiosensitizing mechanism (induced by electron attachment release of the iodide anion coupled to the formation of the reactive 5-yl radical) suggested by refs. 32 and 33.
